# Bat Bites and Rabies PEP in the Croatian Reference Centre for Rabies 1995–2020

**DOI:** 10.3390/v16060876

**Published:** 2024-05-30

**Authors:** Radovan Vodopija, Ivana Lojkić, Daniela Hamidović, Jelena Boneta, Dora Primorac

**Affiliations:** 1 Department of Epidemiology, Andrija Štampar Teaching Institute of Public Health, 10000 Zagreb, Croatia; radovan.vodopija@stampar.hr (R.V.); dora.primorac@stampar.hr (D.P.); 2 Laboratory for Rabies and General Virology, Croatian Veterinary Institute, 10000 Zagreb, Croatia; 3 Ministry of Environment and Green Transition, 10000 Zagreb, Croatia; daniela.hamidovic@mingor.hr; 4 Institute of Public Health of Zagreb County, 10290 Zaprešić, Croatia; jelena.boneta@zzjz-zz.hr

**Keywords:** bat bite, rabies virus, Zagreb, bat lyssaviruses, post-exposure prophylaxis (PEP), pre-exposure prophylaxis (PrEP), bat monitoring

## Abstract

Seroprevalence of lyssaviruses in certain bat species has been proven in the Republic of Croatia, but there have been no confirmed positive bat brain isolates or human fatalities associated with bat injuries/bites. The study included a retrospective analysis of bat injuries/bites, post-exposure prophylaxis (PEP) and geographic distribution of bat injuries in persons examined at the Zagreb Antirabies Clinic, the Croatian Reference Centre for Rabies. In the period 1995–2020, we examined a total of 21,910 patients due to animal injuries, of which 71 cases were bat-related (0.32%). Of the above number of patients, 4574 received rabies PEP (20.87%). However, for bat injuries, the proportion of patients receiving PEP was significantly higher: 66 out of 71 patients (92.95%). Of these, 33 received only the rabies vaccine, while the other 33 patients received the vaccine with human rabies immunoglobulin (HRIG). In five cases, PEP was not administered, as there was no indication for treatment. Thirty-five of the injured patients were biologists or biology students (49.29%). The bat species was confirmed in only one of the exposure cases. This was a serotine bat (Eptesicus serotinus), a known carrier of *Lyssavirus hamburg*. The results showed that the bat bites were rather sporadic compared to other human injuries caused by animal bites. All bat injuries should be treated as if they were caused by a rabid animal, and according to WHO recommendations. People who come into contact with bats should be strongly advised to be vaccinated against rabies. Entering bat habitats should be done with caution and in accordance with current recommendations, and nationwide surveillance should be carried out by competent institutions and in close collaboration between bat experts, epidemiologists and rabies experts.

## 1. Introduction

The last case of human rabies in the Republic of Croatia was recorded in 1964 [1], and since then, there have been only two imported cases of human rabies, one in 1989 and the other in 1996, both in persons arriving from neighboring Bosnia and Herzegovina. Sylvatic rabies persisted, however, appearing in waves, affecting the entire territory with the exception of coastal parts and islands [2,3]. In Croatia, oral rabies vaccination (ORV) campaigns targeting Red Foxes (*Vulpes vulpes*) and Golden Jackals (*Canis aureus moreoticus*) were launched in 2011 and were and still are carried out twice a year, in spring and autumn [4,5]. A few years after the start of the program, the first results began to show, with a steady decrease in rabies in foxes, other wildlife, and domestic animals. The last known case of rabies in wildlife, the Red Fox, was recorded in 2014 [4], and there have been none since. In 2021, the European Commission (EU) officially declared Croatia a rabies-free country with Commission Implementing Regulation EU 2021/620 [6].

Human rabies, a vaccine-preventable zoonosis, is very rare in Europe. On the contrary, dog-mediated rabies continues to pose a threat in the European part of Turkey, as stray dog populations are high and measures to control dog populations are only partially successful [7]. Although sylvatic rabies has been successfully eliminated in most European Union (EU) countries, new cases of rabies have emerged in recent years in previously rabies-free areas in Poland and, more recently, in Hungary and Slovakia [8,9,10]. The illegal importation of pets into Europe has emerged as a new potential threat, as has been observed in France [11]. Apart from these illegal activities, the current potential rabies threats in Europe are imported cases, e.g., in travelers, and bat rabies.

Rabies is caused by negative-strand RNA viruses belonging to the genus Lyssavirus, family Rhabdoviridae of the order Mononegavirales. Although it is completely preventable, it is responsible for an estimated 59,000 human fatalities annually, mainly in sub-Saharan Africa and Southeast Asia [12]. The World Organization for Animal Health (WOAH) has set the year 2030 as the deadline for its eradication [13].

According to the current International Committee on Taxonomy of Viruses (ICTV Release 2021) [14], the genus *Lyssavirus* consists of 17 species, which are divided into three phylogroups. Phylogroup I includes *Lyssavirus rabies* (Rabies virus, RABV), *Lyssavirus duvenhage* (Duvenhage virus, DUVV), *Lyssavirus hamburg* (European bat 1 lyssavirus, EBLV-1), *Lyssavirus helsinki* (European bat 2 lyssavirus, EBLV-2), *Lyssavirus bokeloh* (Bokeloh bat lyssavirus, BBLV), *Lyssavirus australis* (Australian bat lyssavirus, ABLV), *Lyssavirus aravan* (Aravan virus, ARAV), *Lyssavirus khujand* (Khujand virus, KHUV), *Lyssavirus irkut* (Irkut virus, IRKV), *Lyssavirus formosa* (Taiwan bat lyssavirus, TWBLV) and *Lyssavirus gannoruwa* (Gannoruwa bat lyssavirus, GBLV). Phylogroup II species are *Lyssavirus lagos* (Lagos bat virus, LBV), *Lyssavirus mokola* (Mokola virus, MOKV) and *Lyssavirus shimoni* (Shimoni bat virus, SHIBV). Tentative phylogroup III includes *Lyssavirus caucasicus* (West Caucasian Bat Virus, WCBV), *Lyssavirus ikoma* (Ikoma Lyssavirus, IKOV) and *Lyssavirus lleida* (Lleida Bat Lyssavirus, LLEBV), while *Kotalahti Bat Lyssavirus* (KBLV) and *Matlo Bat Lyssavirus* (MBLV) have not yet been classified. Although RABV is responsible for the vast majority of rabies cases in humans, all lyssaviruses can cause fatal encephalitis in both humans and other mammals. Current rabies vaccines provide partial or complete protection against phylogroup I lyssaviruses but no effective protection against phylogroup II and III lyssaviruses [15].

Currently, there are 1460 living bat species worldwide [16]. They are the second largest order of mammals and make up about 20% of all classified mammal species worldwide [17,18]. Globally, the order Chiroptera currently comprises 21 families and 234 genera [16]. The order is typically divided into two suborders, Megachiroptera (megabats, fruit bats) and Microchiroptera (small bats), but has been reclassified as Yinpterochiroptera and Yangochiroptera. The Yinpterochiroptera consist of the lineage formerly classified as megabats and the microbat family Rhinolophidae, while the Yangochiroptera consist of the remaining three microbat lineages: Emballonuroidea, Noctilionoidea and Vespertilonoidea [19]. Of the ~1460 different bat species worldwide, 45 exist in the EU, clustered into five families [20]. With as many as 34 recorded bat species, Croatia is one of the countries with the greatest biodiversity in Europe [21,22,23]. All bat species are strictly protected, and it is not justified under the EU Habitat Directive to kill them to determine rabies status if they are caught [24]. According to UNEP/EUROBATS Agreement Resolution 5.2 Bats and rabies in Europe, bat rabies should be monitored throughout the scope of the Agreement along with a number of precautionary measures and conservation of bats [25]. Croatia ratified the UNEP/EUROBATS Agreement on the Conservation of European Bat Populations in 2000 [26].

Six lyssaviruses are reported to be circulating in Europe: EBLV-1, EBLV-2, BBLV, WCBV, LLEBV and KBLV [27,28]. Four of the viruses, EBLV-1, EBLV-2, BBLV and KBLV, are associated with European bats of the Vespertilionidae family [27,28]. The other two lyssaviruses, WCBV and LLEBV, are associated with bats from the family Miniopteridae [27,28], specifically with Schreiber’s Bent-winged Bat (*Miniopterus schreibersii*), a migratory species. Of all six lyssaviruses, only EBLV-1 and EBLV-2 have caused rabies in humans [29,30], and more than 90% of bat rabies cases have been associated with the serotine bat (*Eptesicus serotinus*) infected with EBLV-1 [31,32]. EBLV-1 has also been detected in sheep [33], cats [34] and Stone Marten (*Martes foina*) [35].

According to Rabies Bulletin Europe [36], 1725 bats were tested in 2022, and 23 of them, originating from five European countries (France, Germany, Poland, the Netherlands and Switzerland) were lyssavirus-positive.

According to the 2019 annual epidemiological report on rabies published by the European Centre for Disease Prevention and Control (ECDC) [37], EU/EEA Member States reported five human lyssavirus infections that year. Four travel-associated human rabies cases were reported by Italy, Latvia, Spain and Norway, with exposures occurring in Tanzania, India, Morocco and the Philippines, respectively. One locally acquired fatal case of EBLV-1 infection was reported from France [37].

To date, several active and passive bat rabies surveillance programs have taken place in the Republic of Croatia; the largest survey was conducted in 2016–2017. Although there were no proven lyssavirus-positive isolates, anti-EBLV-1 antibodies were found in 5.71% of bats, all from continental Croatia [38].

The preferred vaccines for rabies pre-exposure prophylaxis (PrEP) and post-exposure prophylaxis (PEP) are Human Diploid Cell Culture Rabies Vaccines (HDCV) and Purified Chick Embryo Cell Culture Vaccine (PCECV). The World Health Organization (WHO) guidelines for PrEP and PEP for human rabies [39] categorizes bat injuries/exposures as No. III exposure risk, requiring immediate wound cleansing and prompt administration of rabies vaccine and human rabies immunoglobulin (HRIG). It is a worrying fact that the rabies vaccines available for PrEP and PEP are not effective against phylogroup II and III lyssaviruses [15,40]. Therefore, Evans et al. emphasize the need for the development of a universal pan-lyssavirus rabies vaccine that could successfully prevent human rabies in all circumstances [41]. In the following, we provide an overview of individuals admitted to the Zagreb Antirabies Clinic (ARC) from 1995 to 2020 due to bat injuries/bites, taking into account that human–bat interactions pose a serious risk to human health. We also present a list of all bat species that occur in the Republic of Croatia and the greater Zagreb area.

## 2. Methods

### 2.1. Data Collection and Study Design

The Ethics Committee of the Andrija Štampar Teaching Institute of Public Health has approved this retrospective data analysis with the subjects involved. Informed consent was obtained from all subjects and/or their legal guardians.

In this study, all cases of bat bites in humans from 1995 to 2020 at the Zagreb ARC were examined. This period was chosen without prior knowledge of whether cases of bite injuries were reported at the beginning of the study period (1995–2000). Data were collected from the official patient registry of Zagreb ARC for the entire study period. The data included the following parameters: patient’s age and sex, occupation, animal species involved in the incident, veterinary examination of the biting animal (if any), location of the wound, type of treatment received, previous vaccination, hospitalization and geographic location of the place where the bat bite occurred. From the records, the following variables were considered: occupation, type of treatment and geographic location where the bat bite occurred. The retrospective analysis of the data aims to show the frequency of bat injuries/bites, PEP and geographical distribution of affected bat injuries of individuals examined at Zagreb ARC.

### 2.2. Statistical Analyses

We calculated the frequency of bat bites in different occupational groups and the event rates for exposed occupational groups (biologists) and inhabitants of Zagreb. To estimate the frequency of bat bites among biologists, the mean number of biology students enrolled during the study period was estimated (http://www.pmf.unizg.hr/oldwww/osiguravanje_kvalitete/studenti accessed on 16 February 2024). To estimate the frequency of bat bites in the wider Zagreb area during the study period, we estimated the mean population size of the city in the period 1995–2020. To account for all sources of uncertainty, confidence intervals were calculated for event rates per occupational group [42]. For the confidence interval between two rates, the “test-based method” is used [43]. The *p*-value is determined using the Chi2 statistic.

## 3. Results

**Figure 1:**
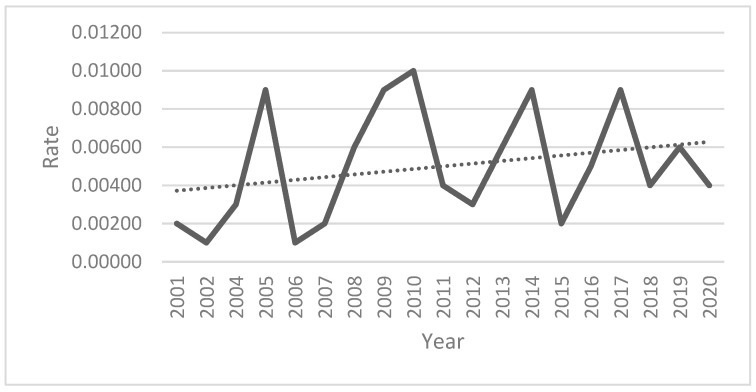
Trends of the incidents of reported bat bites.

According to Zagreb ARC records, a total of 21,910 people were examined for animal bites between 1995 and 2020, of whom 4574 (20.87%) received a PEP against rabies (Table 1). Of this number, 71 bat injuries were registered in the period 2001–2020. No bat bites were reported at the beginning of the period studied (1995–2000), but we have nevertheless decided to include this period in the analysis and discuss possible reasons for this. The trendline shows a slight increase in the number of bat bites compared to the total number of bites and a decrease in the total number of bites (Figure 1).

Of the total 71 patients with bat bites, 66 patients (92.96%) received PEP. Five patients did not receive PEP for the following reasons: two bats were proven lyssavirus negative at the Laboratory for Rabies and General Virology Croatian Veterinary Institute Zagreb; in two other cases, there was no indication for PEP, and in one case, the animal that caused the injury could not be identified, i.e., the injury could not be attributed to bats. However, only in one case was the bat species determined. In 2009, a biologist was bitten by a serotine bat in the Sunger cave near the town of Delnice (Primorsko-goranska County) (Table 2). The patient received a full course of PEP with a rabies vaccine and HRIG and was subsequently proven to be free of the disease.

Of the 66 patients who received PEP following exposure to bats, 11 were biologists and 23 were biology or science students who were exploring caves across the country to study bats as part of their work or studies (N = 35) (Table 2). Overall, 49.29% of the injured patients belonged to this profession. The remainder were members of various other professions such as veterinarians, veterinary students and a forestry worker or people who came into contact with bats by chance (Table 3). All biologists and biology students (except the one previously vaccinated biologist) received PEP with the rabies vaccine and the HRIG, while patients in the other group received PEP with the vaccine only. We calculated the frequency of bat bites in different occupational groups.

From the above results, we identified a group with the highest risk of bat bite, namely the biologists with a total bite rate of 23.65 (1.75 annually). We also calculated the bite rate of Zagreb residents bitten in Zagreb and the wider Zagreb area (Medvednica mountain and Veternica cave) (N = 42). Most of the bites occurred in the greater Zagreb area, the rest in caves and nature/national parks throughout the country. According to WHO guidelines, PEP is administered after almost every documented contact (Table 2). The calculated bite rate per study period for greater Zagreb area is 0.0525 (Table 4).

**Figure 2:**
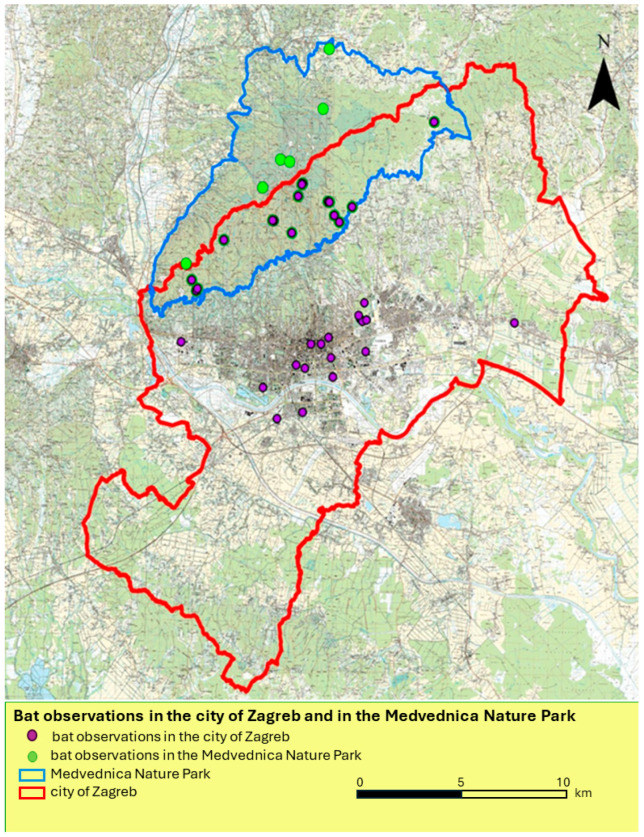
Bat occurrence in the city of Zagreb and in the Medvednica Nature Park. The nature park is located in the north of the city of Zagreb; the map was created according to [22,44].

According to the data on bat occurrence in Croatia since 1995 (Figure S1, Table S1) [22,23], all bat species identified as carriers of bat lyssaviruses in Europe have been detected there. In the greater Zagreb area, 24 species have been recorded, of which 16 species roost in the Veternica Cave in the Medvednica Nature Park immediately north of the city of Zagreb [22,44] (Figure 2, Table 5).

## 4. Discussion

Of the six lyssaviruses circulating in insectivorous bats in Europe, EBLV-1 and EBLV-2 are responsible for most cases of bat rabies in Europe. EBLV-1 is detected in the serotine bat and sometimes also in its sibling species *Eptesicus isabellinus* [31] and is responsible for more than 90% of all bat rabies cases [45] and human fatalities [46]. The geographical distribution of the serotine bat covers the whole of Europe with the exception of Ireland, Norway, Finland and Estonia. Outside Europe, it is found in Turkey, across the Middle East, the Caucasus, Central and Southeast Asia, China and Korea [47,48]. EBLV-2 has been isolated from the Daubenton’s bat (*Myotis daubentonii*), which is distributed in most parts of Europe up to the Ural region in the Russian Federation and along the Black Sea to northern Kazakhstan, and from the pond bat (*Myotis dasycneme*), whose geographic range extends from northern France and southern Sweden across central Europe to the Siberian region of the Russian Federation and also reaches Bosnia and Herzegovina and the southern border of Romania [16,48,49,50,51,52]. Other bat lyssaviruses, WCBV [53], LLEBV [54,55], BBLV [46] and KBLV [56,57], are occasionally isolated in Europe and are associated with Schreiber’s Bent-winged Bat, Natterer’s Bat (*Myotis nattereri*) and Brandt’s Bat (*Myotis brandtii*).

WCBV and LLEBV, the phylogroup III lyssaviruses that are not covered by vaccine protection, have been detected in a number of specimens of Schreiber’s Bent-winged bat, a species that is widespread in Croatia and is associated with subterranean roosts. In Croatia, its current population is estimated at around 60,000 individuals; it is endangered (EN) and is in an unfavorable conservation status [22]. According to the International Union for Conservation of Nature (IUCN), it has vulnerable (VU) status; in many EU countries, it is in an unfavorable conservation status and is geographically distributed from southwestern Europe across the Mediterranean, the Balkans, the Carpathians and the Caucasus [22,58]. It is a seasonal long-distance migrant [58,59], and according to current knowledge, bat experts should handle it with caution. Other viruses such as Lloviu virus (LLOV) [60,61] and various alphacoronaviruses have also been detected in this species in Croatia [62] and neighboring countries, so occasional surveillance of this species is recommended. To date, LLBV has been detected in two Schreiber’s bent-winged bats in Spain and France [54,55], and WCBV has been detected in a cat in Italy [53].

Bat monitoring usually uses protocols for active and passive surveillance based on the recommendations of the First International Conference “Rabies in Europe”, Kyiv, Ukraine, 2005 [63] and EUROBATS Resolution 5.2 [25]. Active surveillance (proactive surveillance) is based on the monitoring of free-living native bat populations for Lyssavirus infections. Oral swabs, saliva, feces, guano or blood samples can be used for lyssavirus surveillance [38,64]. The focus of research can either be on screening of all abundant bat species or on monitoring high-risk bat species in specific areas. In passive surveillance (re-active surveillance), sick, suspected rabies-infected or dead bats of all species are screened for lyssaviruses. All dead bats, regardless of species, should be submitted to national reference laboratories for lyssavirus testing [63]. In 1955, a lyssavirus was isolated from the Noctule (*Nyctalus noctula*) in the former Yugoslavia, confirming bat rabies [65]. In Croatia, one of the first studies on bat rabies was conducted in 1968 for military purposes [66], and for many years thereafter, bat research was meagre. In 1986, 30 Serotine bats were tested negative for rabies at the Croatian Veterinary Institute using the direct fluorescent antibody (DFA) test [67]. Further research was suspended due to the Croatian War of Independence (1991–1995) and resumed in 2008, when 98 dead bats from six genera (Miniopterus, Myotis, Nyctalus, Rhinolophus, Pipistrellus, Plecotus, Eptesicus and Hypsugo) were collected throughout Croatia and tested as rabies negative [68]. From 1986 to 2016, approximately 600 bats tested negative for lyssavirus using the DFA test and/or RT-PCR.

These results are reflected in the practice of the Zagreb ARC, as bats are not considered a health problem. Between 1995 and 2000, not a single person sought medical attention due to bat exposure. Although no bat bites were reported during this period, we consider it important to show this for several reasons. Firstly, at that time, Croatia was affected by sylvatic rabies, with the red fox being the main reservoir and vector and thus the most frequently diagnosed rabid animal. The percentage of rabies-positive foxes was up to 23.6 between 1977 and 2010 [4]. Secondly, Croatia has only been a member of the Agreement on the Conservation of Populations of European bats (EUROBATS) since 2000, and it was only then that extensive campaigns began with the aim of raising public awareness of the need and importance of protecting bats due to the global decline in their populations, but also of the danger of bat rabies. For the reasons given above, it does not mean that there were no bites if no cases of bites were reported in the period 1995–2000. From 2001 onwards, the number of patients who had contact with bats increased slightly, and by the end of 2020, there were a total of 71 examined and 66 treated patients for bat injuries (Table 1 and Table 2). Although the trendline shows a fluctuation in incidence rates between years (Figure 1), it is obvious that it is a very small number (1–8) of injured persons on an annual basis. With the exception of five patients, all were residents of the city of Zagreb or its surroundings. Half of the above patients (49.29%) were biologists or biology students who had been injured during their field work while handling bats in caves or working with mist nets, mostly in protected areas (Table 2). In the years when most contacts with bats were recorded (Table 2), the victims belonged to the most exposed group (biologists), and we can assume that some kind of bat fieldwork was organized for biology students in these years. All of them were immunized with a full course of antirabies vaccine and HRIG. However, contacts with bats in the Zagreb region are relatively rare (overall rate = 0.0525) and are usually associated with certain occupational groups such as biologists and biology students (rate = 23.65) (Table 3 and Table 4). Other patients who came to Zagreb ARC were accidentally bitten/injured by a bat, usually during work such as roof repair, attic repair, hotel/apartment renovation or outdoor activities. There have also been cases where a bat has accidentally flown into a bedroom at night, and such cases are not uncommon and have been described previously [69]. This category of patients only received PEP with a rabies vaccine. Five patients were from outside the Zagreb region, but still preferred to seek help at the Zagreb ARC rather than at the regional epidemiological centers. The reason for this could be the patients’ trust in the Zagreb clinic, but also the lack of awareness of some physicians who do not share the opinion that bat species in Croatia pose a rabies risk. Their opinion was wrong, especially in the light of UNEP/EUROBATS Resolution 5.2 [25] and the bat species recorded in Croatia (Table S1 and Table 5, Figure S1). According to these data, all bat species identified as carriers of bat lyssaviruses in Europe, such as the Serotine bat, carrier of EBLV-1, Daubenton’s bat, carrier of EBLV-2, Schreiber’s Bent-winged Bat, carrier of WCBV and LLEBV, Natterer’s bat and the Pipistrelle Bat, carrier of BBLV, the Brandt’s bat, carrier of KBLV and Noctule, are present in Croatia. All these bat species roost in the Veternica Cave and in the greater Zagreb area and are widely distributed in Croatia [22,70].

In 2016–2017, Šimić et al. conducted active surveillance of bat populations in continental and Mediterranean parts of Croatia to determine the prevalence of individual species and their potential impact on public health. In this comprehensive work, 440 bats from seven species (*E. serotinus*, *Myotis blythii*, *Myotis emarginatus*, *Myotis myotis*, *M. nattereri*, *Min. schreibersii* and *Rh. ferrumequinum*) and 11 sites were tested, and the presence of EBLV-1 antibodies was confirmed in three 5.71% of the bats tested [38]. However, the serology was specific for EBLV-1, so the presence of other lyssavirus species in Croatia cannot be excluded. Similar studies on active surveillance of bats were conducted in the neighboring countries of Slovenia and Serbia, but neither virus-neutralizing antibodies nor viruses have found [71,72]. In contrast, EBLV-1-positive Serotine bats were regularly found in neighboring Hungary [73].

Today, the number of rabies cases in non-flying mammals has drastically decreased thanks to the implementation of national rabies programs in Europe, mainly through the use of ORV in wild animals [74]. Although it is very rare, and only six fatal cases have been reported since 1977, the risk of rabies transmission from bats exists not only for bat handlers but also for ordinary citizens [48,75]. All persons whose profession is closely related to working in nature, such as biologists or biology students, veterinarians or veterinary students, speleologists or forest workers, should be aware of the risks and get vaccinated to prevent rabies transmission from bats [25]. However, preventive rabies vaccination is not compulsory for science and biomedical students, with the exception of veterinary students. Public awareness and education are strongly recommended, as all bat species in Croatia are in unfavorable–bad (U2) or unfavorable–inadequate conservation status (U1), so a bat bite or scratch should be considered a potential exposure and treated for rabies [22,25,39]. In Croatia, mandatory PrEP against rabies was introduced in 2005, and since then, no bat expert, volunteer or bat rehabilitator has tested positive for rabies, which can be transmitted by bats.

We must emphasize once again that modern concentrated and purified HDCV and PCECV rabies vaccines cannot protect against WCBV and LLEBV [15], the lyssaviruses transmitted by Schreiber’s Bent-winged Bat, an endangered and cave-dwelling bat species in Croatia. Although all bats tested so far at the Croatian Veterinary Institute in Zagreb have been negative, the risk of lyssavirus transmission to humans from bats appears to be low and extremely rare, with bat experts and bat conservationists potentially being the most at risk.

## 5. Conclusions

Although the presence of Lyssavirus has been confirmed in the bat population in Croatia, there have been no positive cases of bat brain isolates or bat-related deaths in humans. Although bat bites are sporadic in the daily work of ARC Zagreb, they can pose a potential threat to humans if not treated according to WHO recommendations. The proportion of patients who received post-exposure prophylaxis (PEP) after a bat injury or bite was very high (92.95%), with bat experts and biology students being the most frequently represented. In Europe, including Croatia, the risk of rabies transmission from bats to humans is extremely low, but due to the presence of lyssaviruses of phylogenetic groups I and III, special precautions should be taken when in contact with bats. Therefore, we need to follow the recommendations of the WHO and EUROBATS Resolution 5.2 and work with bat researchers to establish a surveillance network and raise awareness of the potential threat and the need for continuous and improved surveillance of bat rabies.

## Figures and Tables

**Table 1:** An overall number of patients examined and vaccinated at the Zagreb ARC due to potential rabies exposure in the period 1995–2020. Bat injuries are listed in a separate column.

Year	Overall Number of Patients		Patients with Bat Injuries	
	Examined	Vaccinated	Examined	Vaccinated
1995	1380	187	0	0
1996	1300	196	0	0
1997	1195	203	0	0
1998	1058	182	0	0
1999	1129	195	0	0
2000	1010	168	0	0
2001	878	159	2	2
2002	956	154	1	1
2003	889	127	0	0
2004	902	112	3	3
2005	865	133	8	5
2006	802	172	1	0
2007	811	150	2	2
2008	787	242	5	5
2009	761	227	7	7
2010	664	207	8	8
2011	688	180	3	3
2012	716	218	2	2
2013	685	238	4	3
2014	702	192	6	6
2015	650	145	1	1
2016	630	127	3	3
2017	697	148	6	6
2018	674	176	3	3
2019	622	182	4	4
2020	459	154	2	2
Total	21,910	4574	71	66
Percentage (%)		20.87		92.96

**Table 2:** Geographical location of bat incidents in Croatia and patients’ occupation.

Year	Patients with Bat Injuries		Geographic Locations Where Injuries Took Place	Patients by Profession
	Examined	Vaccinated		
1995	0	0	0	0
1996	0	0	0	0
1997	0	0	0	0
1998	0	0	0	0
1999	0	0	0	0
2000	0	0	0	0
2001	1	1	Zagreb	retiree
	1	1	Miljacka Cave, Oklaj	biologist
2002	1	1	Zagreb	biology student
2003	0	0	0	0
2004	1	1	Pleternica	biologist
	1	1	Veternica Cave	biology student
	1	1	Papuk mountain	biologist
2005	1	1	Zagreb	veterinary student
	1	0 *	Zagreb	tourist
	1	1	Veternica Cave	biologist
	1	1	Zagreb	pupil
	1	1	Zagreb	pupil
	1	0 **	Zagreb	unemployed
	1	1	Zagreb	housewife
	1	0 **	Zagreb	hotel caretaker
2006	1	0 *	Zagreb	hotel manager
2007	1	1	Veternica Cave	biology student
	1	1	Zagreb	economist
2008	1	1	Krupa River	biology student
	1	1	Paklenica National Park	biology student
	1	1	Paklenica National Park	biology student
	1	1	Paklenica National Park	biology student
	1	1	Paklenica National Park	biology student
2009	1	1	Paklenica National Park	biology student
	1	1	Paklenica National Park	biology student
	1	1	Paklenica National Park	biology student
	1	1	Dragina Cave/Odra	biologist
	1	1	Zagreb	biology student
	1	1	Veternica Cave	biology student
	1	1	Sunger Cave ^1^, Delnice	biology student
2010	1	1	Veternica Cave	biology student
	1	1	Veternica Cave	biology student
	1	1	Medvednica Nature Park	biologist
	1	1	Medvednica Nature Park	biologist
	1	1	Medvednica Nature Park	forestry worker
	1	1	Medvednica Nature Park	biologist
	1	1	Paklenica National Park	biology student
	1	1	Bezdanka Cave, Zrmanja	biology student
2011	1	1	Dugi Otok	biologist
	1	1	Zagreb	physician
	1	1	Slunj	worker
2012	1	1	Veternica Cave	child
	1	1	Tounjčica Cave, Tounj	cameraman
2013	1	1	Lasinja	retiree
	1	1	Zagreb	electrician
	1	0 ***	Zagreb	optician
	1	1	Brijuni National Park	painter
2014	1	1	Zagreb	engineer
	1	1	Perušić	biology student
	1	1	Sesvete	electrician
	1	1	Zagreb	biology student
	1	1	Zagreb	lawyer
	1	1	Crnopac Cave, Gračac	biologist
	1	1	Zagreb	architect
2015	1	1	Zagreb	economist
2016	1	1	Zagreb	car painter
	1	1	Sesvete	retiree
	1	1	Vrbovec	biology student
2017	1	1	Zagreb	cameraman
	1	1	Zagreb	student
	1	1	Veternica Cave	biologist
	1	1	Zagreb	electrician
	1	1	Dugi otok	biology student
	1	1	Zagreb	biology student
2018	1	1	Zagreb	retiree
	1	1	Zagreb	retiree
	1	1	Jastrebarsko	trainer in physical educat.
2019	1	1	Zagreb	profession unknown
	1	1	Plitvice Lakes National Park	electrician
2020	1	1	Zagreb	veterinarian
	1	1	Zagreb	student
	1	1	Žumberak mountain/Cave	biology student
Total	71	66		

*—bat tested lyssavirus negative; **—no indication for PEP; ***—the cause of the injury has not been identified. ^1^—bat species identified, Serotine bat.

**Table 3:** Frequency of animal bites in different groups.

Occupation	Biologists	Vets/Forestry Workers	Private Businessmen	Tourists	Children	Retiree	Others
Frequency, n (%)	35 (49.29)	3 (4.22)	15 (21.13)	1 (1.40)	4 (5.63)	5 (70.42)	8 (11.27)

**Table 4:** Bite rates in two groups.

Group	Bite Rate per 1000	95% CI	Incidence Rate Ratio
Biologists	23.650	0.016–0.032	450.45
Persons injured in the greater Zagreb area	0.0525	0.000038–0.000071	(95% CV = 279.20–722.69) *p* < 0.0001

**Table 5:** Bat species in the city of Zagreb, Medvednica Nature Park and Veternica Cave.

Bat Species in the Greater Zagreb Area and Medvednica Nature Park, 24 Species (1995–2018)	Bat Species in Veternica Cave, 16 Species (1995–2018)
*Barbastella barbastellus*	*Barbastella barbastellus*
*Eptesicus serotinus*	*Eptesicus serotinus*
*Hypsugo savii*	
*Miniopterus schreibersii*	*Miniopterus schreibersii*
*Myotis bechsteinii*	*Myotis bechsteinii*
*Myotis blythii*	*Myotis blythii*
*Myotis brandtii*	
*Myotis daubentonii*	*Myotis daubentonii*
*Myotis emarginatus*	*Myotis emarginatus*
*Myotis myotis*	*Myotis myotis*
*Myotis mystacinus sensu lato*	*Myotis mystacinus sensu alto*
*Myotis nattereri*	*Myotis nattereri*
*Nyctalus leisleri*	
*Nyctalus noctula*	*Nyctalus noctula*
*Pipistrellus kuhlii*	
*Pipistrellus nathusii*	
*Pipistrellus pipistrellus*	
*Pipistrellus pygmaeus*	
*Plecotus auritus*	*Plecotus auritus*
*Plecotus austriacus*	
*Plecotus macrobullaris*	*Plecotus macrobullaris*
*Rhinolophus euryale*	*Rhinolophus euryale*
*Rhinolophus ferrumequinum*	*Rhinolophus ferrumequinum*
*Rhinolophus hipposideros*	*Rhinolophus hipposideros*

## Data Availability

All data generated during this study are included in this published article.

## Supplementary Materials

The following supporting information can be downloaded at: https://www.mdpi.com/article/10.3390/v16060876/s1, Table S1. Bat species in Croatia. Figure S1. Data on bat occurrences in Croatia since 1995.
