# Supplementary material for: Bat Bites and Rabies PEP in the Croatian Reference Centre for Rabies 1995–2020
Source: Viruses. 2024 May 30;16(6):876. doi: 10.3390/v16060876 (PMC11209127; doi:10.3390/v16060876)
Supplement: Supplementary file 1 [file viruses-16-00876-s001.zip › viruses-3014241-supplementary.pdf]

Table S1. Bat species in Croatia.

| |
|---|
| **Order Chiroptera - bats** |
| **Rhinolophidae, family** |
| Blasius' Horseshoe Bat (*Rhinolophus blasii*) |
| Mediterranean Horseshoe Bat (*Rhinolophus euryale*) |
| Greater Horseshoe Bat (*Rhinolophus ferrumequinum*) |
| Lesser Horseshoe Bat (*Rhinolophus hipposideros*) |
| **Vespertilionidae, family** |
| Western Barbastelle Bat (*Barbastella barbastellus*) |
| Northern Bat (*Eptesicus nilsonii*) |
| Serotine Bat (*Eptesicus serotinus*) |
| Savi's Pipistrelle Bat (*Hypsugo savii*) |
| Alcathoe Whiskered Bat (*Myotis alcathoe*) |
| Lesser Mouse-eared Bat (*Myotis blythii*) |
| Brandt's Bat (*Myotis brandtii*) |
| Long-fingered Bat (*Myotis capaccinii*) |
| Pond Bat (*Myotis dasycneme*) |
| Daubenton's Bat (*Myotis daubentonii*) |
| David's mouse-eared bat (*Myotis davidii*) |
| Geoffroy's Bat (*Myotis emarginatus*) |
| Greater Mouse-eared Bat (*Myotis myotis*) |
| Whiskered Bat (*Myotis mystacinus*) |
| Natterer's Bat (*Myotis nattereri*) |
| Greater Noctule Bat (*Nyctalus lasiopterus*) |
| Leisler's Noctule Bat (*Nyctalus leisleri*) |
| Noctule (*Nyctalus noctula*) |
| Kuhl's Pipistrelle Bat (*Pipistrellus kuhlii*) |
| Nathusius'Pipistrelle Bat (*Pipistrellus nathusii*) |
| Common Pipistrelle Bat (*Pipistrellus pipistrellus*) |
| Soprano Pipistrelle (*Pipistrellus pygmaeus*) |
| Brown Big-eared Bat (*Plecotus auritus*) |
| Grey long-eared Bat (*Plecotus austriacus*) |
| Balkan Long-eared Bat (*Plecotus kolombatovici*) |
| Alpine Long-eared Bat (*Plecotus macrobullaris*) |
| Particoloured Bat (*Vespertilio murinus*) |
| Miniopteridae - family |
| Schreiber's Bent-winged Bat (*Miniopterus schreibersii*) |
| **Mollosidae, family** |
| European Free-tailed Bat (*Tadarida teniotis*) |

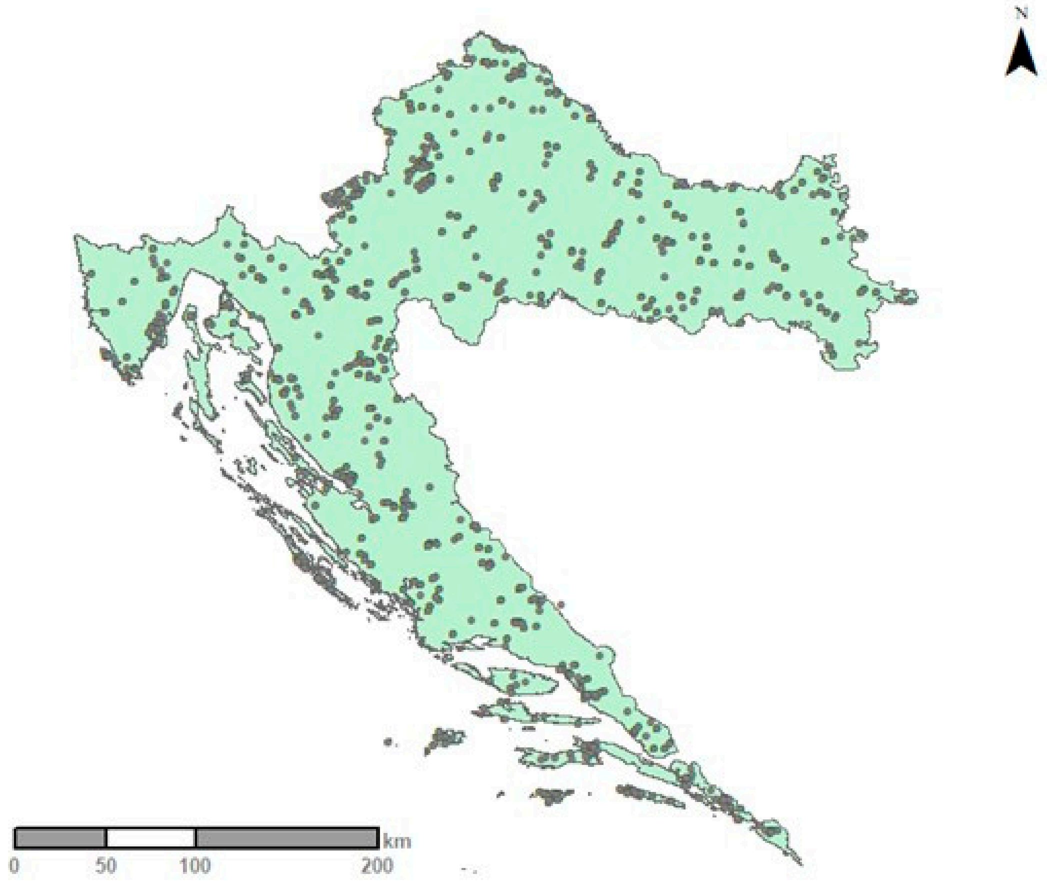


Figure S1. Data on bat occurrences in Croatia since 1995.
